# A novel synthetic nucleic acid mixture for quantification of microbes by mNGS

**DOI:** 10.1099/mgen.0.001199

**Published:** 2024-02-15

**Authors:** Hailing Wanghu, Yingzhen Li, Jin Huang, Kangze Pu, Fengming Guo, Peiwen Zhong, Ting Wang, Jianying Yuan, Yan Yu, Jiachang Chen, Jun Liu, Jason J. Chen, Chaohui Hu

**Affiliations:** ^1^​ Guangzhou KingCreate Biotechnology Co., Ltd., Guangzhou, Guangdong, 510005, PR China; ^2^​ Changsha KingMed Diagnostics Group Co., Ltd., Changsha, Huna, 410000, PR China; ^3^​ KingMed School of Laboratory Medicine, Guangzhou Medical University, Guangzhou, Guangdong, 511436, PR China

**Keywords:** internal-standards, microbial quantification, mNGS

## Abstract

Metagenomic next-generation sequencing (mNGS) provides considerable advantages in identifying emerging and re-emerging, difficult-to-detect and co-infected pathogens; however, the clinical application of mNGS remains limited primarily due to the lack of quantitative capabilities. This study introduces a novel approach, KingCreate-Quantification (KCQ) system, for quantitative analysis of microbes in clinical specimens by mNGS, which co-sequence the target DNA extracted from the specimens along with a set of synthetic dsDNA molecules used as Internal-Standard (IS). The assay facilitates the conversion of microbial reads into their copy numbers based on IS reads utilizing a mathematical model proposed in this study. The performance of KCQ was systemically evaluated using commercial mock microbes with varying IS input amounts, different proportions of human genomic DNA, and at varying amounts of sequence analysis data. Subsequently, KCQ was applied in microbial quantitation in 36 clinical specimens including blood, bronchoalveolar lavage fluid, cerebrospinal fluid and oropharyngeal swabs. A total of 477 microbe genetic fragments were screened using the bioinformatic system. Of these 83 fragments were quantitatively compared with digital droplet PCR (ddPCR), revealing a correlation coefficient of 0.97 between the quantitative results of KCQ and ddPCR. Our study demonstrated that KCQ presents a practical approach for the quantitative analysis of microbes by mNGS in clinical samples.

## Data Summary

Our sequencing data has been uploaded to the Sequence Read Archive (SRA) under the accession number PRJNA998608. Additionally, other supporting data including the software, databases and algorithms used in the biosignature analyses, were detailed in the Methods, and the primer sequences used in PCR were included in the Supplementary Materials.

**Impact Statement**

While mNGS has been used in clinical diagnosis of infectious diseases, the lack of precise and reproducible quantitative measurement has been a limitation. We have now developed an innovative approach that enables quantitative analysis of microbes in clinical specimens. We believe this advancement would promote the application of mNGS not only in clinical science but also in environmental science.

## Introduction

Over the past decade, the incidence of infectious diseases has ascended globally with sepsis fatality rates as high as 50 % [1]. Additionally, the emerging and re-emerging infectious diseases, infections with multiple microbes, and cases of fever with unknown origins pose significant threats to global health [2]. Early identification of the corresponding pathogen is crucial for enabling targeted therapy, which substantially increased patient survival rates [3]. Consequently, innovation and optimization of pathogen detection strategy with molecular technology has been one of the research focuses.

Pathogen detection technologies could be broadly categorized into culture-based and non-culture-based methods [4, 5]. As a non-culture-based technology, mNGS is distinguished by its unbiased nature and wide detection range [6]. Moreover, since mNGS analyses all microbes in a specimen, it exhibits higher sensitivity than traditional detection techniques, particularly in dealing with rare or previously unknown pathogens, such as the identification of the SARS-CoV-2 genome [7]. Although mNGS has been successfully applied in diverse clinical applications including infections of the bloodstream, central nervous system and respiratory tracts, some challenges remain [8–11].

For instance, the inability of mNGS to accurately quantify targets arises from the absence of a universal reference standard for pathogen determination in clinical diagnosis [12]. Moreover, variations in DNA sheer sizes and the diversity of human genome material within clinical samples further affect the interpretation of the sequencing results. These factors collectively contribute to variations in the positive predictive value (PPV) and negative predictive value (NPV) of molecular assays in clinical diagnosis [13–15].

To establish a reliable and universal reference standard in high-throughput sequencing, a variety of approaches have been developed, including spike-in controls, either using the microbial genome sequence known in nature but absent from the test sample [16], or employing commercial plasmids as a reference standard for NGS [17]. Furthermore, unique fragments of microbial genomes have been used as a direct internal reference for quantifying target abundance [18]. Apart from the diverse designs of spike-in controls, there were studies that addressed applications in different fields including a recent approach aimed at monitoring and assessing microbe risks in the environment through mNGS [19]. Some investigations have focused on the quantification of 16S or ITS amplicon sequences [20, 21]; however, these approaches are tailored for bacteria or fungal microbes, and differ from the targets of mNGS. While quantitative studies based on amplicon sequencing have made progress by designing and incorporating 16S, 18S and ITS fragments [20] or directly correlating amplicon sequencing with ddPCR for quantification [21], neither of them might be readily applied to mNGS. This is because mNGS directly sequences all the nucleic acid (NA) in the sample including host DNA. Therefore, there is a need for the development of microbial quantification methods specifically for clinical applications using mNGS.

In this study, we introduce a novel method for the absolute quantification of microbes in clinical mNGS analysis. A total of 15 synthetic dsDNA molecules were meticulously designed and prepared as spike-in controls. These molecules were divided into five groups, each comprising three molecules that form a concentration gradient. These spike-in molecules were subsequently added to NA extracted from clinical samples as an internal standard (IS) and go through library preparation and sequencing. An IS-regression curve could be obtained by using a mathematical model in this study to convert microbial reads to their copy numbers.

## Methods

### Design and synthesis of IS

For the convenience of synthesis and the compatibility with library preparation, the size range of IS was determined to be 1000 bp with a GC content of 35–70 % and a consecutive base number of less than four. Adhering to these criteria, DNA fragments were randomly generated and 50-nt consecutive contigs were extracted from each fragment to align with nucleotide sequences in NCBI databases. Subsequently, 15 sequences were meticulously screened based on their minimum homology to any sequences available in NCBI databases. These fragments were synthesized by Sangon Biotech Co., Ltd (Shanghai, China), and ligated to pUC57 and transformed into DH5α competent *E. coli* which resulted in 15 IS-clones. The authenticity of the IS was confirmed by Sanger sequencing. Following these, 15 pairs of PCR primers (Table S2) were designed to amplify the IS sequences from each clone, generating 15 target fragments of 970±10 bp. The integrity of the IS fragments was confirmed by sequencing, and their concentrations were determined with a Qubit 4.0 fluorometer. The 15 IS fragments were then mixed at equal ratios and diluted to five concentration gradients of 2×10^−1^, 2×10^−2^, 2×10^−3^, 2×10^−4^, and 2×10^−5^ ng/μL, which is available from KingCreate with the part of KS647-KRDmN-48

### Metagenomic control material and clinical specimens

Two mock microbial genomic materials that simulating mixed metagenomic bacteria and fungal, MSA-4000 and MSA-1010 respectively, were procured from ATCC (Manassas, VA). In addition, 36 clinical specimens including oral swabs, blood, cerebrospinal fluid (CSF), and bronchoalveolar lavage fluid (BALF), were generously provided by KingMed Diagnostics, Inc. (Guangzhou, China), which was approved by the Ethics Commission of KingMed Diagnostics, Inc.

To replicate samples with different ratios of human host DNA to microbial DNA, DNA extracted from human leukaemia T cells was used as human host background. The quantities of microbial DNA (MSA-4000 or MSA-1010) and human DNA were 5.5 ng human DNA and 1.1 ng microbial DNA in the low human background group, and 5.0 ng human DNA with 0.01 ng microbial DNA in the high human background group.

### DNA extraction, library preparation and sequencing

DNA was extracted from specimens with TIANamp Micro DNA Kit (TIANGEN, Beijing, China). The NA concentration was determined with a Qubit fluorometer. For library preparation, 10 ng of specimen NA were mixed with 2 µL of IS to make the NGS library using the kit of KS619-DNAmN24 (KingCreate, Guangzhou, China). Briefly, sample NA was treated with the enzyme mixture in the KS619-DNAmN24 kit and the resulting fragments were analysed with an automatic NA protein analyser Qseq-100 (Bioptic, Jiangsu Province, China). The fragments used for library preparation were within the range of 250~350 bp.

Sequencing was performed on the Illumina NextSeq 550Dx platform, generating an average data amount of 40 GB per sequencing run. The data acquisition was conducted through single-end sequencing with a read length of 75 bp.

### Bioinformatic analysis

The extensive sequence data generated by mNGS was first processed by bcl2fastq software (v2.20.0.422) to demultiplex sequencing data and to convert base calling files into raw fastq-format files. Then Fastp (version 0.23.1) was used to remove adapters, low-quality sequences and duplicated sequences. The resulting data was aligned to human reference (hg38) using BWA (0.7.17-r1188, Burrow-Wheeler Aligner) and reads originated from human were filtered. Subsequently, the filtered sequences were compared to those in RefSeq (Release 211, March 2022) and an internally constructed database with IS reference sequences, the remaining high-quality sequences were annotated with microbial classifications such as bacteria, fungal, viral, and parasites along with the IS. Statistical analysis was performed to quantify the results for each microorganism with the IS. A self-developed process (https://github.com/kcmngs/ISQ) was utilized to pre-configure the IS results across different gradients with a linear regression model, which was used to plot of sequence versus concentration. The corresponding microorganisms were incorporated into the scatter plot formula to calculate their concentrations, and were ultimately converted to copy numbers.

To assess the reproducibility of quantitative data based on IS calculation at different dataset sizes, the raw data of a sample at 180M was subjected to random extractions with seqkit (version 0.15.0) at dataset sizes of 1M, 5M, 20M, 35M, 50M sequentially. Each extraction was repeated 20 times to minimize sampling bias.

### ddPCR

The fluorescent dye method was employed for ddPCR with BIO-RAD QX200. The experiments followed the EvaGreen Supermix protocol provided by the manufacturer (BIO-RAD, Hercules, CA). This approach facilitated the accurate quantification of target DNA and further validated the results obtained by the KCQ method.

## Results

### The workflow and mathematical model

**Fig. 1.**
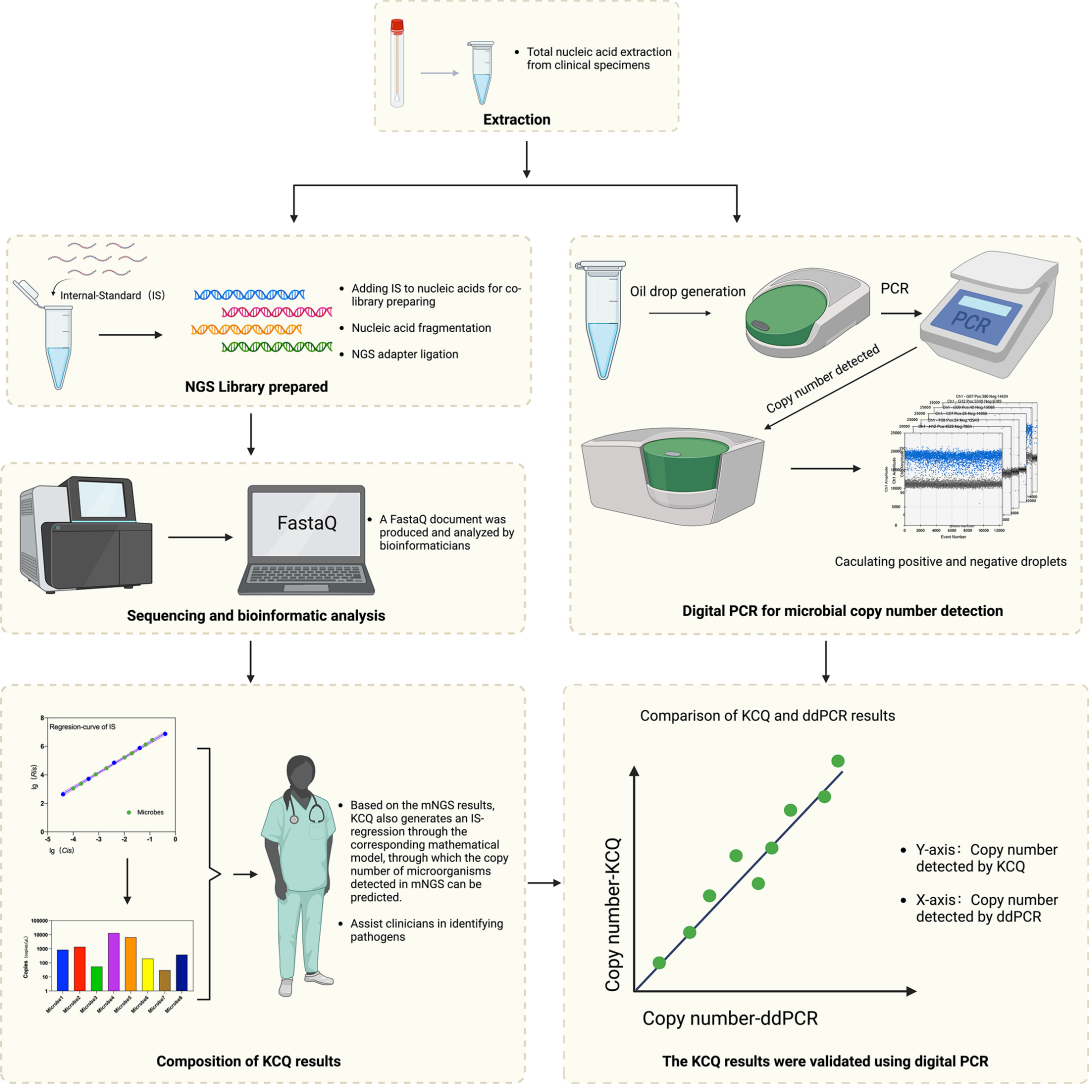
Validation of quantitative mNGS by KCQ assay with that of ddPCR. Total nucleic acid was extracted from samples and was added to reactions along with IS. The library was then prepared and sequenced by NGS. Bioinformatics analysis is able to obtain the IS sequences captured during the sequencing process. An IS regression-curve is prepared for the quantitation of microbial copy numbers before a quantitative mNGS test report is generated. Parallely, the same NA was used in ddPCR assay and the resulting copy numbers were compared to those obtained by KCQ.

In the standard clinical mNGS workflow, total NA is extracted from specimens, and a library is prepared before sequencing [22–24]. Bioinformatic analysis is then conducted to screen and interpret the sequence data. The results are integrated into a comprehensive test report to assist physicians in evaluating the potential etiological significance for the patient (Fig. 1).

The synthetic IS developed in this study does not alter the mNGS process. Instead, the IS is added during the library preparation, which facilitates the generation of a regression-curve in the post-sequencing bioinformatic analysis through our mathematical models (see below). The regression-curve enables absolute quantification of all microbes in the sample, which offers insights into the inputs and copies of all potential targets in the clinical specimen. The quantitative capability of KCQ is ultimately validated by the comparison of the reads produced by KCQ and those obtained by ddPCR (Fig. 1).

The mathematical model of KCQ is established based on the relationship between dsDNA reads and dsDNA inputs in high-throughput sequencing. Through Pearson correlation analysis [25], we observed a robust linearity (r^2^ ≥0.99) between the inputs of IS (*C*is) and the number of IS sequences detected (*R*is). This strong correlation suggests a parallel linearity between the detected microbial reads (*R*mic) and the microbe input (*C*mic) in clinical samples, expressed by Equation 1.

(1) $$\frac{C_{\text{is}}}{R_{\text{is}}}=\frac{C_{\text{mic}}}{R_{\text{mic}}}$$

To generate an IS regression-curve and establish a reference standard for mNGS, the IS should consist of synthetic dsDNA at various concentration gradients. To cover the quantitative range of microbes (10~10^6^ cfu/mL) in clinical applications, we prepared five IS concentration gradients from 2×10^−1^ to 2×10^−5^ ng/µL, each consisting of three different IS molecules. Therefore, a total of 15 highly specific synthetic dsDNAs, grouped in sets of three, were created, resulting in five concentration groups. Given the substantial order of magnitude difference in IS reads detected among the gradients, the IS sequence detection amount for each gradient and their corresponding inputs were meticulously recorded before plotting the regression curves (Fig. 1).

To enhance the quantification of the test results and allow them to be validated by widely used assays such as qPCR and ddPCR, it is necessary to convert the mass concentrations to molecular copy numbers [26] based on the mass of microbe (*C*mic), their genome size (*L*mic) and unit DNA mass (*DNA*c), as described by Equation 2.

(2) $${\text{COPY}}_{\text{mic}}=\frac{C_{\text{mic}}}{L_{\text{mic}}\cdot {\text{DNA}}_{c}}$$

Compared to the results of conventional high-throughput sequencing, which only provides microbial reads in samples, the KCQ can further offer the copy numbers of microbes in the sample. This capacity enables absolute quantification of microbes.

### The quantitative performance of KCQ

To evaluate the quantitative capability of the KCQ system, the mock microbial genomic material MSA-4000, which includes DNA from ten different Gram-positive and Gram-negative bacteria, was analysed by KCQ. MSA-4000 was accompanied with DNA copy numbers of each microorganism determined with ddPCR by ATCC. Furthermore, to investigate the quantitative ability of KCQ under different amounts of microbial NA, experiments were carried out at constant IS input (2 µL) and three different MSA-4000 inputs at 1 µL, 2 µL, and 5 µL, respectively. Vice versa, the experiment was then repeated at constant MSA-4000 input (5 µL) and three different IS inputs (2 µL, 4 µL, and 6 µL) respectively.

**Fig. 2.**
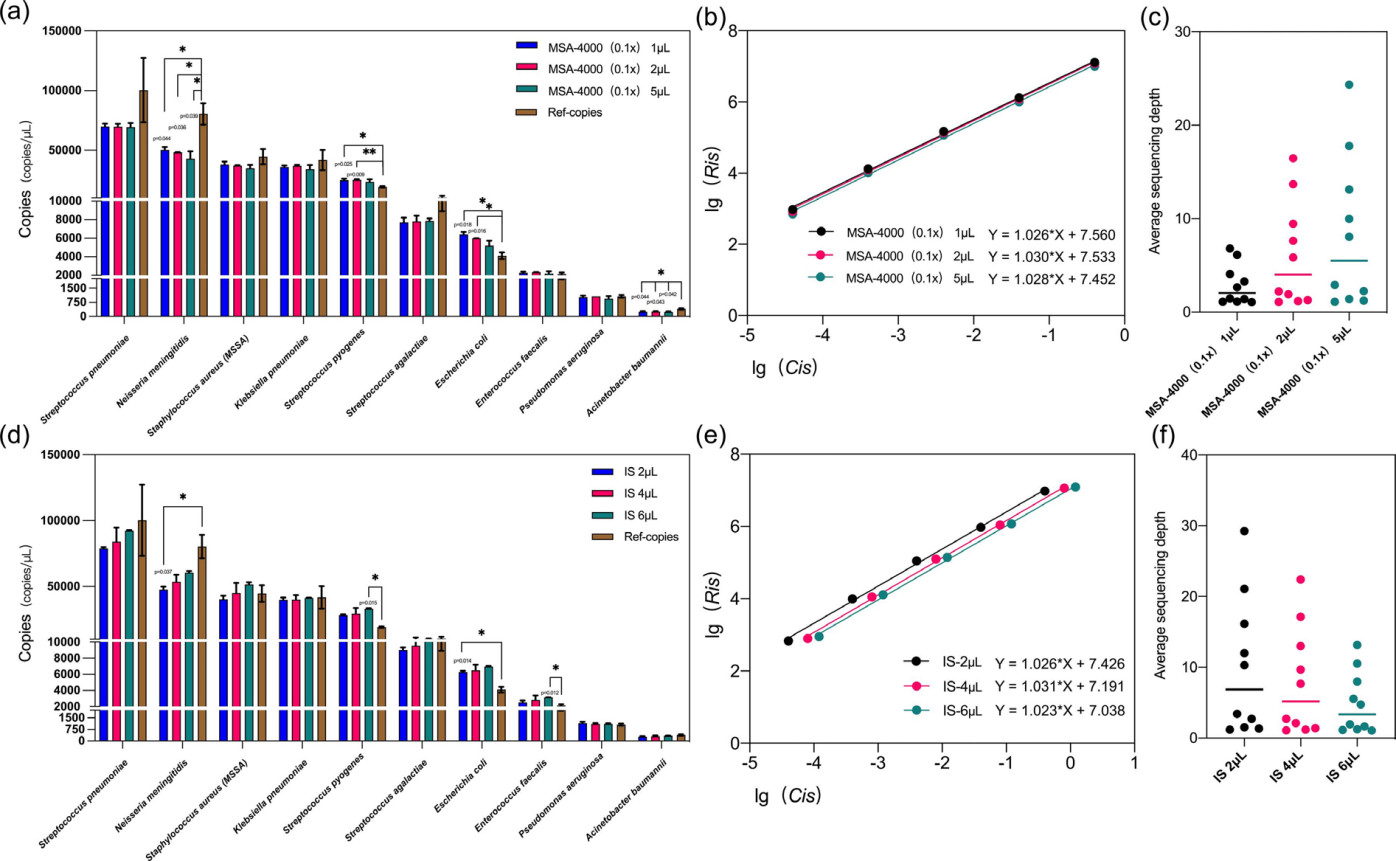
The quantitative accuracy of KCQ. (**a**) The effect of different MSA-4000 inputs on the quantition by KCQ. Ref-copies were copies quantified by ATCC through ddPCR. MSA-4000 0.1 x was prepared by 1 : 10 dilution in the buffer. Different input volumes of 1 µL, 2 µL or 5 µL of the diluted MSA-4000 were added before the library preparation. A t-test analysis of resulting KCQ copy numbers versus Ref-copy numbers was performed, and the groups with *P*<0.05 were labelled with * and those with *P*<0.01 were labelled with **. (**b**) Regression curves of IS under different MSA-4000 input volumes. *Ris*: the number of IS sequences detected, *Cis*: the inputs of IS. (**c**) Average sequencing depth of microorganisms under different MSA-4000 inputs. (**d**) The effect of different IS inputs on the quantitation by KCQ. Then 2 μL, 4 µL or 6 µL of IS were added before the library preparation. A t-test analysis of resulting KCQ copy numbers versus Ref-copy numbers was performed, and groups with *P*<0.05 were labelled with *. (**e**) Regression curves of IS under different IS input volumes. *Ris*: the number of IS sequences detected. *Cis*: the inputs of IS. (**f**) Average sequencing depth of microorganisms under different IS inputs.

The results revealed that at constant IS input, variations in MSA-4000 DNA input did not result in significant differences in microbial sequence reads quantified by the KCQ system, and most microbial targets exhibited copy numbers close to the reference copy number provided by ATCC (*P*>0.05) at Y-axis (Fig. 2a). However, for *Neisseria meningitidis*, *Streptococcus pyogenes*, *Escherichia coli* and *Acinetobacter baumannii*, significant differences were observed (*P*<0.005) between KCQ copy numbers and reference copy numbers of these bacteria by t-test analysis.

Similarly, when maintaining a constant input MSA-4000 DNA, alterations in the IS input did not result in substantial fluctuations in the microbial copy numbers detected by KCQ compared to the reference copy numbers at Y-axis (Fig. 2d). After analysing the raw sequencing data, it was identified that microbes with significant differences were mainly attributed to two factors: 1. Contamination of experimental reagents or the environment [27], such as *Escherichia coli*; 2. The assignment of species reads during bioinformatic analysis is influenced by the species within the same genus or similar species.

Next, we delved into the linear performance of IS in the aforementioned experiments. The analysis showed that changes in MSA-4000 input quantity had no significant impact on the intercept and slope of the IS regression curve (Fig. 2b), suggesting that the input quantity of microbial DNA does not exert a substantial effect on the microbial quantification accuracy of the KCQ system. In contrast, the alteration of IS input quantity did not influence the linearity of the regression curves but did impact the intercept of the regression curve (Fig. 2e). This observation suggests that an increase in IS input would lead to a reduction in microbial reads. This phenomenon is attributed due to the nature of mNGS which randomly sequences all NA in the sample. Therefore, as the data amount from other NA increases, the microbial reads may decrease to some extent, affecting the quantitative ability of KCQ.

In both experiments, the analysis of the average sequencing depth of microorganisms revealed expected trends. The average sequencing depth of microorganisms gradually increased with the rise in MSA-4000 input quantity (Fig. 2c). Conversely, the average sequencing depth of microorganisms decreased with an increase of IS input (Fig. 2f), This observation underscores the importance of optimizing IS input quantity in clinical applications, as discussed further below (see Fig. 5a, b).

**Fig. 3.**
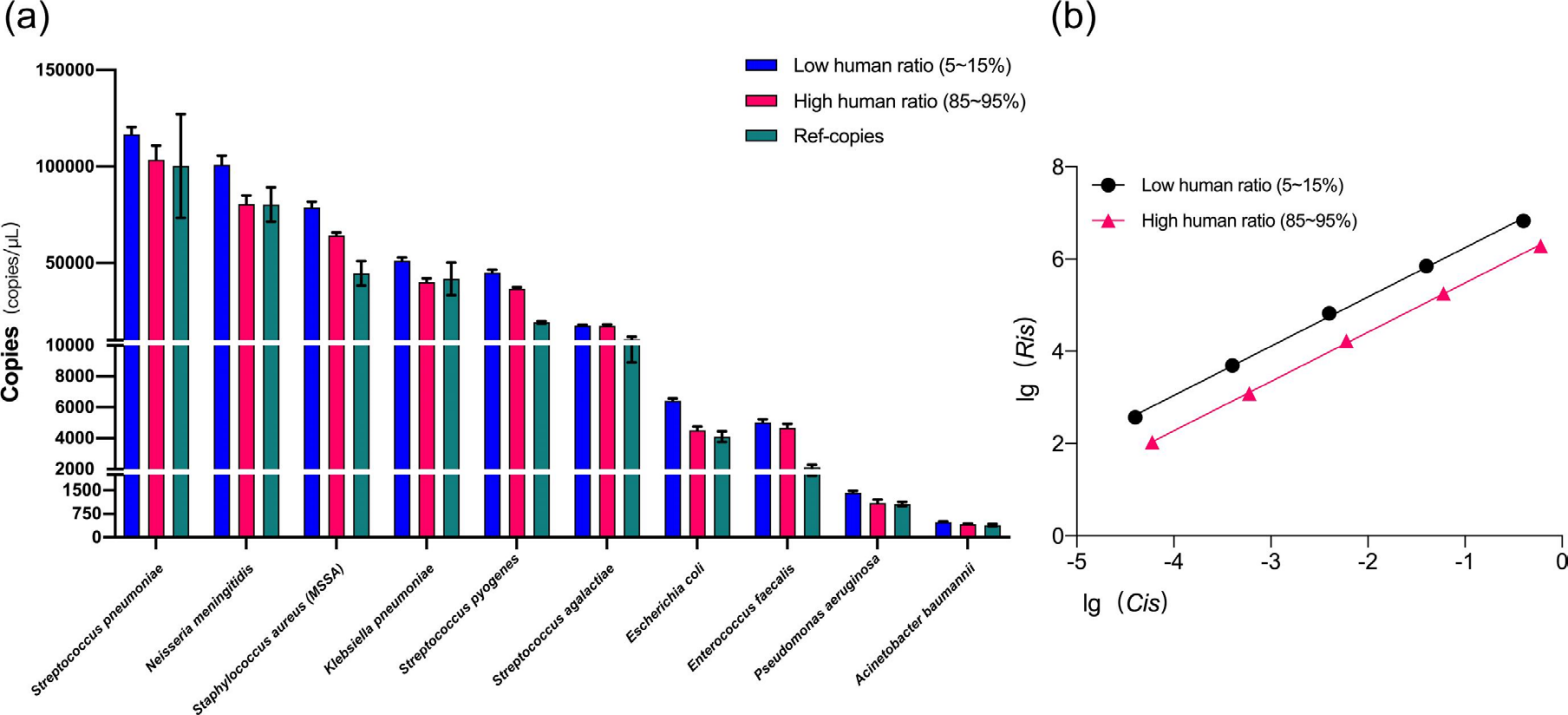
Effect of host background on the quantitation by KCQ. (a) Comparison of microbial copy numbers under low and high host DNA background. Human ratio, the number of human reads in the sequencing results as a proportion of the total data amount of the sample. (b) Regression curves of IS under low and high host background.

### Effect of host background on microbial quantitation by KCQ

To investigate how the quantitative ability of KCQ might be influenced by host background, NA extracted from human leukaemia T cells was used as the anthropomorphic background to analyse the performance of KCQ at different ratios of human cellular DNA to microbial DNA. Samples simulating low (5–15 %) and high (85–95 %) host DNA backgrounds were prepared for NGS through the adjustment of the ratio of human NA to MSA-4000 DNA during the library preparation.

Unexpectedly, the quantitative ability of KCQ showed little impact introduced by the host DNA background, the IS-copies exhibited minimal difference from the reference copies (Ref-copies, Fig. 3a) under both high and low human DNA background. In fact, some bacteria with high copy number, including *Neisseria meningitidis*, *Staphylococcus aureus*, and *Klebsiella pneumoniae*, were more accurately quantified in high NA background (Fig. 3a). Possible reasons for this observation are detailed in Fig. S3, available in the online version of this article.

**Fig. 4.**
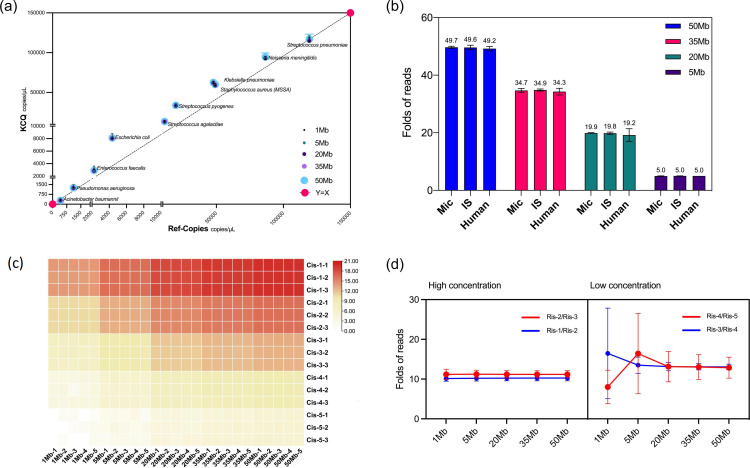
Effect of host background on the quantitation by KCQ. (**a**) Comparison of microbial copy numbers under low and high host DNA background. Human ratio, the number of human reads in the sequencing results as a proportion of the total data amount of the sample. (**b**) Regression curves of IS under low and high host background.

Moreover, the differences in IS regression curves at high and low host NA backgrounds were analysed. It was observed that the background had little impact on both the linearity and the slope of IS regression-curve (Fig. 4b). However, the intercept between the two regression curves was different. We believe that it was attributed to the larger data amount occupied by host NA, leading to a decrease in detected IS reads, even though it did not alter the linearity of the IS (Fig. 3b).

**Fig. 5.**
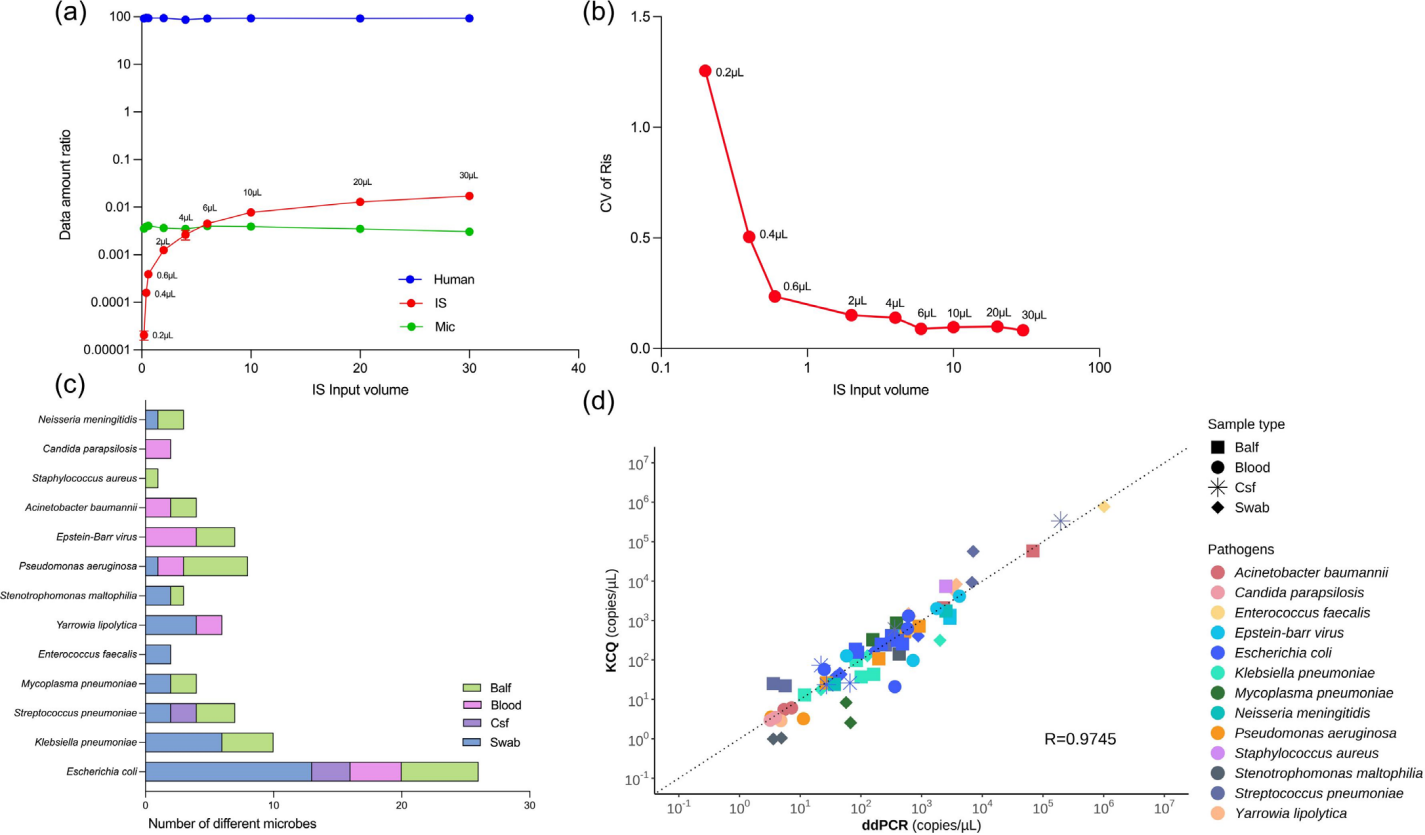
The quantitative capability of KCQ with Cclinical specimens. (a) The effect of IS input amount on the detection of microbial reads in a CFS specimen. (b) Effect of different IS input volumes on the detection of IS’s reads by KCQ. The data used for Cv calculation is the ratio of reads of IS between different gradients (Ris-n/Ris-n-1). (c) Thirty-six clinical specimens was quantitatively analyzedanalysed for the presence of microorganisms by KCQ and by ddPCR respectively. A total of 83 fragments were detected, including those commonly found pathogens such as *Escherichia coli*, *Klebsiella pneumoniae*, *Streptococcus pneumoniae*, *Mycoplasma pneumoniae*, *Enterococcus faecalis*, *Yarrowia lipolytica*, *Stenotrophomonas maltophilia*, *Pseudomonas aeruginosa*, Epstein-barr virus, *Acinetobacter baumannii*, *Staphylococcus aureus*, *Candida parapsilosis*, and *Neisseria meningitidis* among others. (d) Quantitative results were comparable by KCQ and ddPCR.

### The effect of data amount on microbial quantitation by KCQ

In the realm of high-throughput sequencing analysis, data amount plays a pivotal role in quality control alongside sequencing depth, sequencing coverage, and coverage rate. To evaluate the consistency of KCQ quantification under different data amounts, a test sample with a data amount exceeding 50 Mb was selected for a data gradient test. Gradient data sets of 1 Mb, 5 Mb, 20 Mb, 35 Mb, and 50 Mb were repeatedly retrieved from the sample 20 times. The retrieved data were plotted to compare the copy numbers detected by the IS in KCQ (y-axis) to Ref-copies (x-axis). The results, illustrated in Fig. 5a, demonstrate that the copy numbers detected by IS in KCQ closely aligned with Ref-copies for every bacterium. Moreover, the copy numbers at different data amounts exhibit proportional consistency (Fig. 4a). Further analysis of the detected reads from microbe, IS and human background under different data gradients revealed that the fold increase in detected reads from microbe, human and IS remained consistent with the folds increased in data amount (Fig. 4b). In other words, under varying data amounts, the detected reads among the three different populations remained the same. This consistency may explain why the quantitative ability of KCQ remains unchanged under different data amounts.

Since the IS consists of five different molecules at three concentrations, an analysis was conducted on the reads detected from each IS molecule at every concentration under different data amounts to investigate whether the IS sequence detection remains stable under both high and low data amounts. The results presented in Fig. 5c demonstrated that at higher concentrations (Cis-1, Cis-2 and Cis-3), the IS reads detection was remarkably stable at each data amount.

Moreover, the ratio of the detected reads between each gradient of IS under different data amounts was further analysed. As depicted in Fig. 4d, the ratio of IS gradient reads in the range of 35M~50M showed consistency with a CV% ranging from 6–24 %. However, the CV% fluctuated between 6 and 106 % in the range of 1M to 5M, until the data amount reached 20 Mb, at which point the CV% returned to the range of 6–30 %. Therefore, considering both cost and the KCQ stability, it was recommended to use 20 Mb as the standard to perform KCQ analysis in clinical applications.

### Validation of KCQ with clinical specimens and comparison with ddPCR

Since sequencing technology is a primarily qualitive method, it makes it difficult to quantify the pathogens in clinical specimens [28]. Previous studies have employed real-time PCR to differentiate colonizing bacteria from pathogenic bacteria by a defined cut-off value [29, 30]. We aimed to develop a comprehensive quantitative molecular assay, hence the performance of KCQ was investigated with a set of clinical specimens.

While the impact of IS input on quantitative results has been assessed previously (Fig. 2d, f), it was imperative to establish a standardized IS input for quantitative analysis by KCQ in clinical applications. Given the high proportion of human DNA in clinical samples, even with host DNA removal included in the assay, determining the appropriate range for the IS data amount is essential. A gradient input of IS was tested using a CSF specimen with over 95 % DNA from human genome. As shown in Fig. 5a, an IS input of >6 µL started to preempt the pathogens data amount, while an IS input of <1 µL did not produce consistent data (Fig. 5b). To ensure that the amount of data occupied by the IS input was not excessive in the presence of clinical specimens, while still enabling consistent detection of its own sequences, a uniform input volume of 2 µL was selected for all subsequent experiments.

Subsequently, the validation of KCQ was extended to 36 clinical samples including BALF, peripheral blood, CSF and nasopharyngeal swabs. A total of 83 fragments such as *Streptococcus pneumoniae*, *Klebsiella pneumoniae*, Epstein-Barr virus, *Staphylococcus aureus*, and *Pseudomonas aeruginosa* were detected using ddPCR after sequencing (Fig. 5c) with the primers for microbes listed in Table S1. The quantitative accuracy of KCQ in these clinical samples was in line with our expectations, with a correlation coefficient of 0.97 between quantitative results obtained from KCQ and ddPCR (Fig. 5d). This robust correlation underscores the reliability and accuracy of KCQ in quantifying microbial content in diverse clinical samples.

Additionally, tests using MSA-4000 with copy numbers from the manufacturer (Ref-copies) demonstrated high consistency with the Ref-copies (Fig. S2), further validating the accuracy of ddPCR assay used in this study.

### Limitations and future directions

Due to the limited availability of clinical samples, the testing of a broader range of clinical specimens using KCQ has been constrained. Recognizing the diversity and complexity of clinical specimens, we emphasize that our results provide only a preliminary feasibility analysis of KCQ in the clinical setting.

## Discussion

Several microbial quantification methods are presently available, including colony counting, infection titration, qPCR and ddPCR [4]. The first two culture-based methods are considered to be the gold standard for pathogens quantification; however, they are time-consuming and limited to pathogens that can be cultured *in vitro*. In contrast, qPCR and ddPCR are NA-based quantitative methods that allow rapid and accurate quantification of microbial genomes at the molecular level.

Unlike the aforementioned studies, this paper focuses on the clinical application of mNGS, addressing two critical considerations. Firstly, to ensure that spike-in controls do not introduce any microbial sequences to the test procedure. Secondly, to maintain the quantitative accuracy of spike-in controls without compromise, considering the diversity of clinical samples and the possibility of environmental NA contamination. For example, microbial genomes or their fragments in forward or reverse orientation may lead to confusion in data interpretation in some instances [16, 19].

Indeed, in some studies, commercial or custom-made plasmids have been used as spike-in controls for transcription [17]. However, the use of plasmids may introduce potential environmental contamination coming from the plasmid preparation process. In contrast, the IS molecules developed for KCQ do not exist in nature, to our knowledge. These sequences have been specifically designed and synthesized for the purpose of quantification. Additionally, the sequences can be further modified to accommodate different specificity or melting temperature (Tm) requirements.

It is also important to note that while quantitative methods based on amplicon sequencing in previous studies are practicable, PCR inherently introduces bias. The accuracy of any given taxon’s abundance is believed to be impacted by amplification bias [21].

Table 1 summarized the differences among published studies of NGS quantification. We now describe a novel approach, KCQ, which was shown to be eligible for the quantitative evaluation of microbial copy numbers in clinical specimens using high-throughput sequencing. This method offers a reliable reference standard for accurately accessing microbial risk through NGS analysis.

**Table 1.** Comparison of studies related to NGS quantification

Previous studies related to NGS quantification	Internal standard sequence design	Quantitative method	Applications	Possibilities for applications in clinical mNGS	Refs
1	Use of genome sequences of natural species (marine microorganisms)	Relative quantification	Faecal microorganisms/mNGS	The inserted sequence may have the same fragment in the clinical sample	[16]
2	Use of 16S rRNA or ARGs sequences from natural species*	Absolute quantification	Environmental microorganisms/mNGS	The inserted sequence may have the same fragment in the clinical sample	[19]
3	Inverting the genome sequences of natural species	Relative quantification	Environmental microorganisms/mNGS	The inserted sequence may have the same fragment in the clinical sample	[18]
4	Commercial plasmid sequences were used	Relative quantification	Marine microorganism/metatranscriptome	Plasmids may cause contamination in clinical applications	[17]
5	Synthesis based on 16S rRNA with ITS and other primer segment sequences	Absolute quantification	Environmental microorganisms/tNGS†	Methodology based on amplicon sequencing, not applicable to mNGS	[20]
6	No insertion of internal standards (directly combined with ddPCR)	Absolute quantification	Oral and digestive tract microorganisms /tNGS	Methodology based on amplicon sequencing, not applicable to mNGS	[21]
This study	Multiple double-stranded DNA sequences not found in nature were designed and artificially synthesised to be inserted at different concentration gradients	Absolute quantification	Clinical pathogenic microorganisms/mNGS	Sequences designed and synthesised to ensure that there are no duplicates in clinical samples, in addition to the ability to quantify all targets in the sample.	/

*ARGs: antibiotic resistance genes.

†tNGS：targeted Next-Generation Sequencing.

It is worth noting that the focus of our study was primarily on testing microbes in clinical samples at the NA level. The aim of the study was to optimize the variables in the library preparation, NGS, and bioinformatical analysis. Variations brought by NA extraction step were not analysed. In fact, the same NA was used in this study to compare the sequence reads obtained by ddPCR and KCQ separately.

Nevertheless, we assessed the performance of KCQ in the NA extraction stage. Since the internal standard (IS) consisted of short-chain oligonucleotides, the recovery efficiency of short-chain NA was critical to the accurate quantification. To address this issue, two different NA extraction kits were employed. The NA was extracted from the original sample with the supplementation of IS, and microbial detection and quantification was performed by KCQ. The results showed that the inconsistent recovery efficiency of IS by different extraction methods led to a significant quantitative differences compared to the direct addition of IS to the sample NA. Additionally, it is worth noting that adding IS to clinical specimens before NA extraction required much higher IS input quantity to compensate for low extraction recovery. The IS input needs to be at least 20 times more than when added during library construction to normalize the KCQ quantification ability (Fig. S4).

Further discussion is warranted regarding the reagent contamination issue highlighted in the previous section. In our experiments, we observed variations in the levels of background microbial DNA among different brands of reagents. This variability may significantly impact the interpretation of mNGS results, particularly when the microbial DNA belongs to common species found in clinical settings.

Fig. S5 illustrates the detection of background microbial DNA associated with various brands of fragmentases. The results indicate higher background microbial DNA detected when using Fragmentase-B, with some species such as Bombyx mori nuclear polyhedrosis virus being detected at elevated levels. Substituting with Fragmentase-A significantly reduces the background microbial detection, although some residual DNA remained.

In conclusion, KCQ, an NA-based molecular quantitative technology, was confirmed to have a more accurate quantification function for microbes. However, whether it would fully satisfy complex clinical applications remains to be further tested.

## Supplementary Data

Supplementary material 1
