# Supplementary material for: A novel synthetic nucleic acid mixture for quantification of microbes by mNGS
Source: Microb Genom. 2024 Feb 15;10(2):001199. doi: 10.1099/mgen.0.001199 (PMC10926700; doi:10.1099/mgen.0.001199)
Supplement: Supplementary material 1 [file mgen-10-1199-s001.pdf]

A novel synthetic nucleic acid mixture for quantification of microbes by mNGS

SUPPLEMENTARY DATA

The performance of KCQ in fungal NA

Fungi are common pathogens that need to be analyzed in clinical practice, and their large genomes result in the possibility of some differences in sequencing detection. The ATCC mock fungal DNA mixture MSA-1010, consisting of genomic material from 10 different clinically significant mycobacteria, was therefore used to test the quantitative ability of KCQ. As shown in Supplementary Fig 1A, with the increase of MSA-1010 input, the variations among the triplicate tests of every fungus reduced significantly, and their copy numbers determined by IS (2μL in each reaction) tended to be consistent. Four fungal targets in the material were selected for quantitation by ddPCR as the MSA-1010 product did not come with copy numbers from the manufacture. Quantitative analysis by KCQ and ddPCR was performed using one μL each of MSA-1010, respectively, and it showed that both quantitative methods reached similar copy numbers of each mycobacterium (Supplementary Fig 1B), except for *Candida albicans*, the reasons for which remain to be elucidated.

A

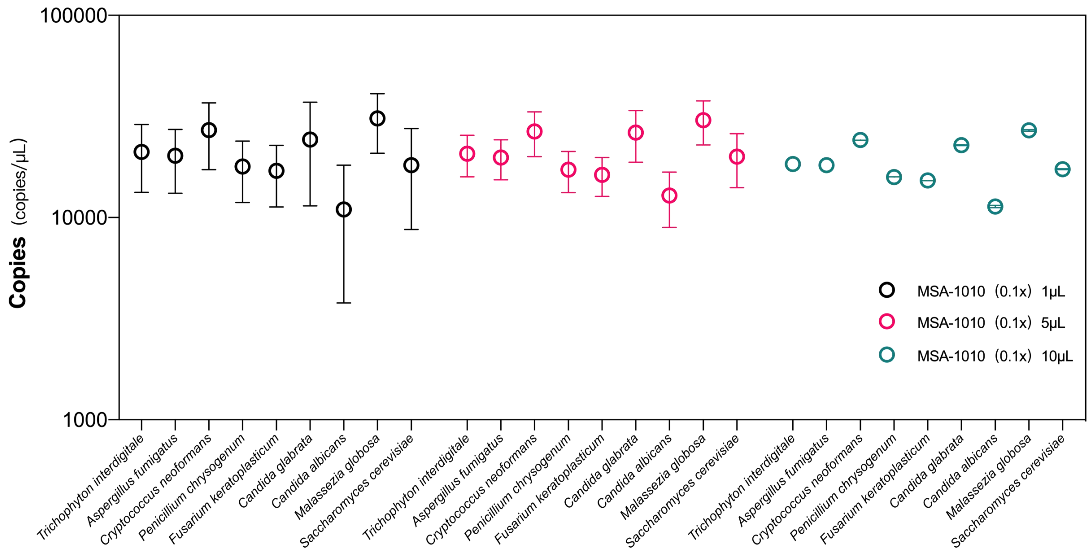

B

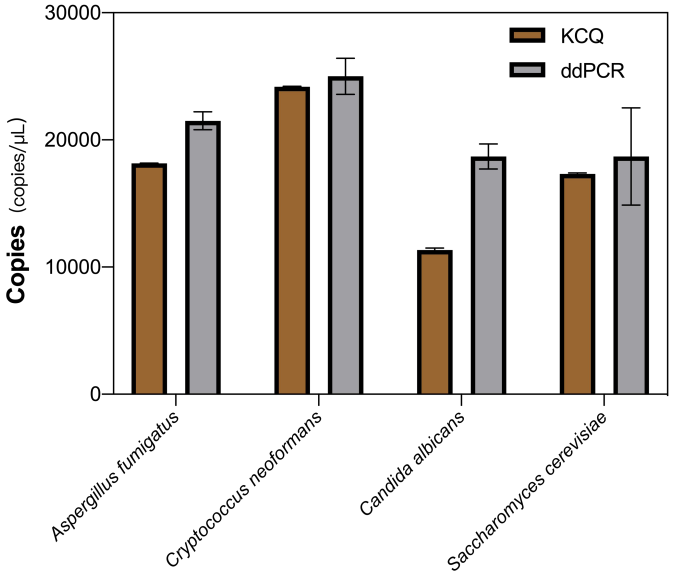

Supplementary Fig 1. Quantitative ability of KCQ for fungi.

(A) The effects of different MSA-4000 input volume on KCQ. With the increase of MSA-1010 input, KCQ quantification results gradually stabilized.

(B) Validation of KCQ results using ddPCR. Four clinically common fungi were selected for ddPCR validation.

Verification of ddPCR accuracy used in this study

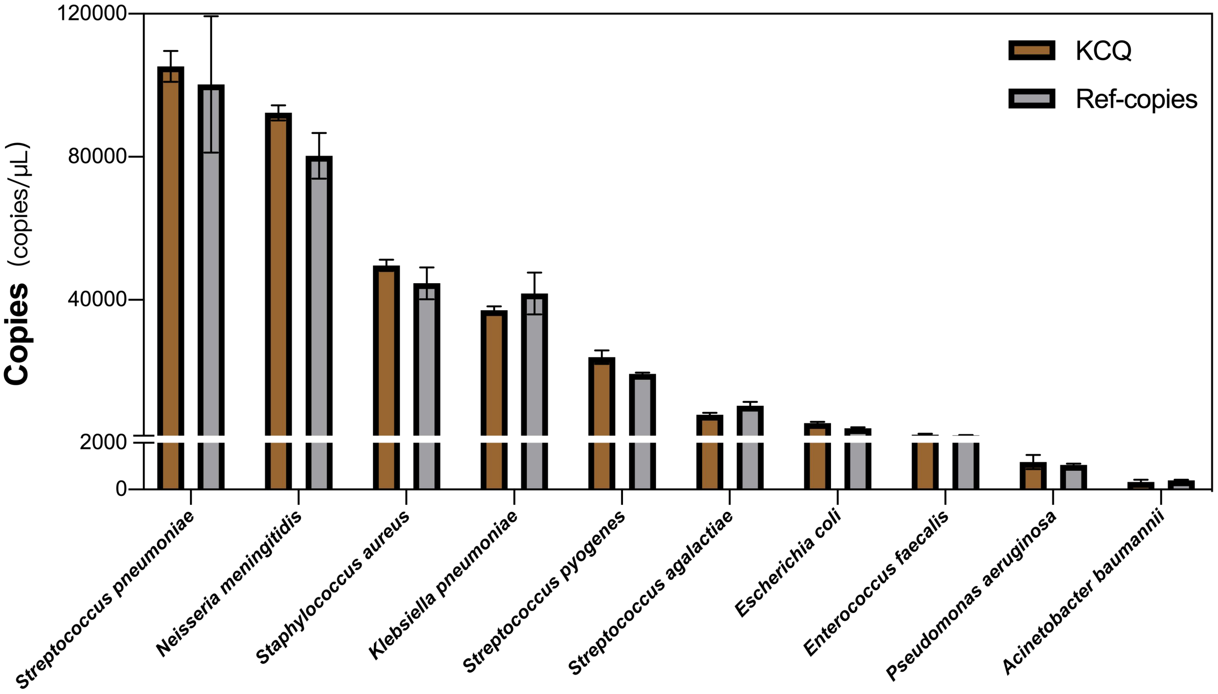

Supplementary Fig 2. Digital droplet PCR instrument Accuracy Verification.

Validation of ten microbial nucleic acid copy numbers in MSA-4000 using our own ddPCR assay

Effect of human background on high copy numbers microbes.

The microbial reads with high copy numbers mentioned in main text were analyzed, and it was found that microbe generally accounted for a small data amount 1.5%~2% under the background of high host ratio. Therefore, the detected microbial reads with high copy numbers or low copy numbers had little fluctuation. However, under low host ratio, the detected microbial reads with high copy numbers accounted for a large data amount 18%~25%, leading to a large fluctuation (Fig. 3C). It was attributed to the fact that fluctuations in microbial reads were narrowed in the high host background (Fig. 3C), while such fluctuations surfaced in the low host background. This could be the major reason for KCQ quantification appears more accurate in sample that has higher human NA background.

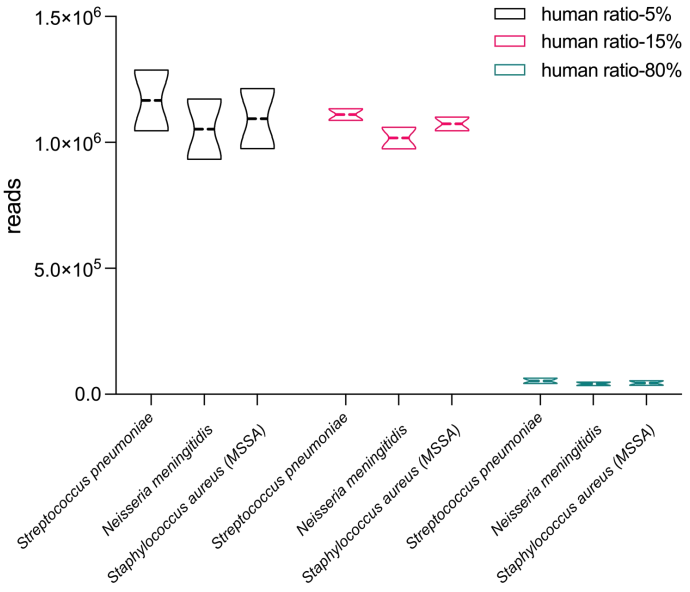

Supplementary Fig 3. Reads of high copy number microbes under different human DNA

Fluctuation of high copy microbe reads under different human ratio. High-copy pathogens fluctuate more in detection when the ratio of human sources is low.

Add IS in the extraction stage

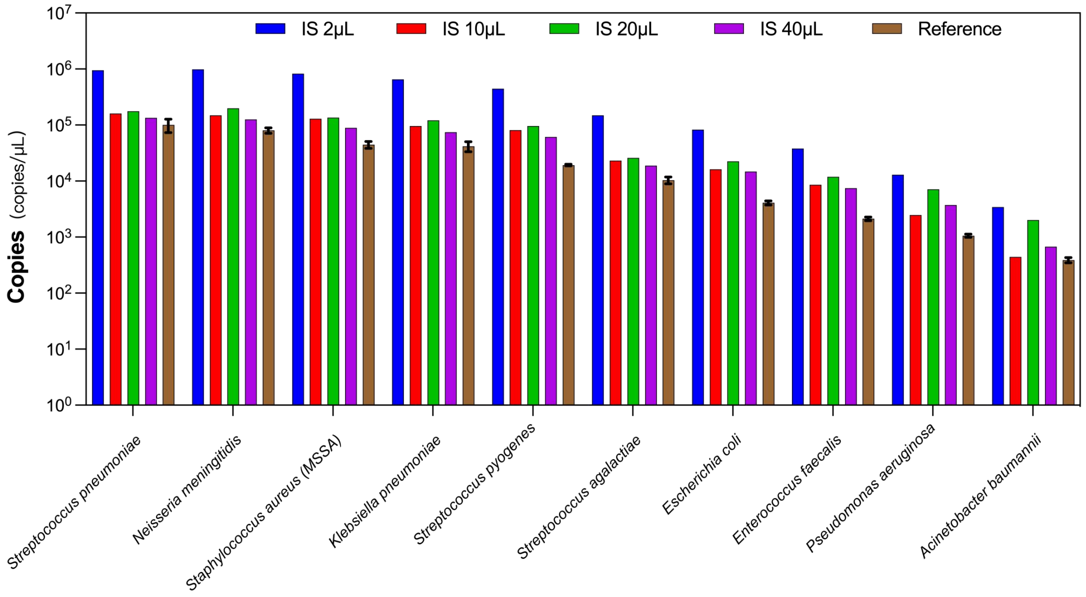

Supplementary Fig 4. Effect of adding different volumes of IS in the NA extraction stage

NA extraction was performed from Balf sample spiked with MSA-4000 and IS. Y-axis: copy numbers. X-axis: The 10 microorganisms included in the MSA-4000. As the volume of IS input gradually increased, the quantitative copy number of each microorganism by KCQ gradually approached the reference copy number provided by ATCC.

Background contamination of reagents in mNGS

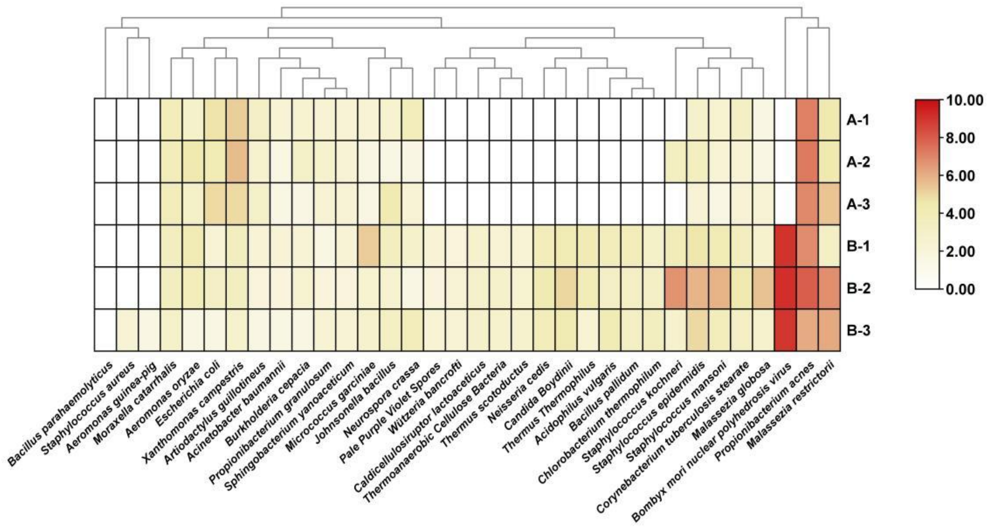

Supplementary Fig 5. Detection of background contamination in different brands of

fragmentation enzymes

A/B：Different brands of fragmentase, Fragmentase-A and Fragmentase-B；1/2/3: Experimental Repeat；Scale：Log(RPM); Most of the background microorganisms were significantly reduced or disappeared when the fragmentase was changed from Fragmentase-B to Fragmentase-A, e.g., *Bombyx mori nuclear polyhedrosis virus*, and there were also some background microorganisms that could not be eliminated, e.g., *Propionibacterium acnes* and *Escherichia coli*, etc.

Table S1. Primers for microorganisms by ddPCR

ddPCR Primers	Sequence (5'- 3')
Acinetobacter baumannii-F	TTCAGCAAGGTCAATCGCTC
Acinetobacter baumannii-R	AAAGGCCAACGTATAGGCAG
Aspergillus fumigatus-F	TTGCTCAAACGATGCTTACA
Aspergillus fumigatus-R	CTATCAAGCGCGAGTTCTAC
Candida albicans-F	TAGCTGAGGCAAGACCAGAT
Candida albicans-R	AGAACATGCTACTGAAGCGG
Candida parapsilosis-F	CAATTGCCATAGCGTGTGTC
Candida parapsilosis-R	GCAGGTAACCATCCACTAGC
Cryptococcus neoformans-F	TTGCGTAGAAAGATCCGGC
Cryptococcus neoformans-R	AGATAAGTCCTAAGACCGCC
Enterococcus faecalis-F	CCGAGTGCTTGCACTCAATTGG
Enterococcus faecalis-R	CTCTTATGCCATGCGGCATAAAC
Epstein-Barr virus-F	CCCAACACTCCACCACACC
Epstein-Barr virus-R	TCTTAGGAGCTGTCCGAGGG
Escherichia coli-F	GGCCTTGCGATGTCTTCAAC
Escherichia coli-R	GTCGAAATGTTCGTCTGCCG
Klebsiella pneumoniae-F	GGCTAAAGCGGTGGGTATTA
Klebsiella pneumoniae-R	TGATATCGGTTAACGCCGTC
Mycoplasma pneumoniae-F	CGTGGTGAAGTGAAACATCTCAG
Mycoplasma pneumoniae-R	AAGCCCTACAACCCCTATCTAATG
Neisseria meningitidis-F	AGCAAAACATCTGGCTGGAA
Neisseria meningitidis-R	TAAATCGGGTACGGCTGTTG
Pseudomonas aeruginosa-F	CTGGGTCGAAAGGTGGTTGTTATC
Pseudomonas aeruginosa-R	GCGGCTGGTGCGGCTGAGTC
Saccharomyces cerevisiae-F	CTGTGACGACCATCAAGGTT
Saccharomyces cerevisiae-R	ACGGCACTTAGTAGGTAGGT
Staphylococcus aureus-F	TTGGTAAGGGTGTCTACAGGG
Staphylococcus aureus-R	CTTGGATACTTTTTCGGCGTC
Stenotrophomonas maltophilia-F	CTGATGCTCAACAAGCCACG
Stenotrophomonas maltophilia-R	CTTCTGCGGACCCGTTTC
Streptococcus agalactiae-F	GGTGGAGATTAATCGGCGTT
Streptococcus agalactiae-R	CTTTGACTTCAAGCATGCGG
Streptococcus pneumoniae-F	ATGCGACTTCTAATTACTCAG
Streptococcus pneumoniae-R	CATTATCAGTCCCAGTCGGT
Streptococcus pyogenes-F	TGCTACTGCAACTGCTCAAA
Streptococcus pyogenes-R	AGCTACCTGCAGAACCACTA
Yarrowia lipolytica-F	TCACTTCGCATTCTCTGGAC
Yarrowia lipolytica-R	GCTCCATCAATCAGTCGGTT

Table S2. Primers for amplification of IS sequences

IS Primers	Sequence (5'- 3')
IS1-1F	GGCGATGTCCCTACCT
IS1-1R	GAGCCTTTGCTGATGC
IS1-2F	ATCCGCCGATGATAGAG
IS1-2R	AAGGGCCATTTGAAGG
IS1-3F	TCAAACGCCAAGGTCA
IS1-3R	GCGAGTAGCCGTTAGGT
IS2-1F	ATACGCCACCACCAAGAT
IS2-1R	AAGACGACTGAACCGAAA
IS2-2F	TCATGCCTACTTTGTTGC
IS2-2R	TATGTGCCGTTATGTGCT
IS2-3F	CGACGCAAGAACACCG
IS2-3R	TCCACTATGACGCACTCC
IS3-1F	ATGTGCCCAGTCCAACG
IS3-1R	CGAGGAAATCCGAGGTG
IS3-2F	TGTATCTGGTAACCCTCG
IS3-2R	TAGTTTCGTCCTTATCTTTG
IS3-3F	GCATTTGCGGTTCTGA
IS3-3R	CTGGAGCAGTCCCTTG
IS4-1F	GCAGGCAAGAAATAGTATCAG
IS4-1R	GGCGAGAAAGCCCAAT
IS4-2F	TCACTCCTGGCTCTTACTC
IS4-2R	TGTGGGCTTATCACTTCTA
IS4-3F	CACGGGTCTATTATTCAACT
IS4-3R	CCTGCTCACGCTCTATC
IS5-1F	TCGCTGTCTCCTGAAATG
IS5-1R	TGCTACCCTCGCAACC
IS5-2F	AGCGTGCTTATGAACTTTG
IS5-2R	CAACCAGGACGGACATT
IS5-3F	GCTCAGACCTCCTATTGT
IS5-3R	CTCACTTGCGTACCTCA
